# Social Media Devices’ Influence on User Neck Pain during the COVID-19 Pandemic: Collaborating Vertebral-GLCM Extracted Features with a Decision Tree

**DOI:** 10.3390/jimaging9010014

**Published:** 2023-01-08

**Authors:** Bassam Al-Naami, Bashar E. A. Badr, Yahia Z. Rawash, Hamza Abu Owida, Roberto De Fazio, Paolo Visconti

**Affiliations:** 1 Department of Biomedical Engineering, Faculty of Engineering, The Hashemite University, Zarqa 13133, Jordan; 2 Department of Electrical Engineering, Al-Ahliyya Amman University, Amman 19328, Jordan; 3 Department of Medical Engineering, Al-Ahliyya Amman University, Amman 19328, Jordan; 4 Department of Innovation Engineering, University of Salento, 73100 Lecce, Italy

**Keywords:** social media usage, smartphones, neck pain, GLCM, decision tree algorithm

## Abstract

The prevalence of neck pain, a chronic musculoskeletal disease, has significantly increased due to the uncontrollable use of social media (SM) devices. The use of SM devices by younger generations increased enormously during the COVID-19 pandemic, being—in some cases—the only possibility for maintaining interpersonal, social, and friendship relationships. This study aimed to predict the occurrence of neck pain and its correlation with the intensive use of SM devices. It is based on nine quantitative parameters extracted from the retrospective X-ray images. The three parameters related to angle_1 (i.e., the angle between the global horizontal and the vector pointing from C7 vertebra to the occipito-cervical joint), angle_2 (i.e., the angle between the global horizontal and the vector pointing from C1 vertebra to the occipito-cervical joint), and the area between them were measured from the shape of the neck vertebrae, while the rest of the parameters were extracted from the images using the gray-level co-occurrence matrix (GLCM). In addition, the users’ ages and the duration of the SM usage (H.mean) were also considered. The decision tree (DT) machine-learning algorithm was employed to predict the abnormal cases (painful subjects) against the normal ones (no pain). The results showed that angle_1, area, and the image contrast significantly increased statistically with the time of SM-device usage, precisely in the range of 2 to 9 h. The DT showed a promising result demonstrated by classification accuracy and F1-scores of 94% and 0.95, respectively. Our findings confirmed that the objectively detected parameters, which elucidate the negative impacts of SM-device usage on neck pain, can be predicted by DT machine learning.

## 1. Introduction

Many social media (SM) users enjoy using many smartphone applications in everyday life and are linked in for a long time to social media such as Facebook, Twitter, LinkedIn, Instagram, Snapchat, Tik Tok, Pinterest, Reddit, YouTube, and WhatsApp. During the browsing routine, users tend to lower their heads and place the smartphone device close to their waist or lap while travelling, such as in a car, bus, or subway train. Using a smartphone for a long time in such a posture will cause a strained spine, neck, and eye fatigue.

Daily, worldwide social media usage in the years 2012–2020 has already been published by the Statista Research Department [1] and the World Health Organization (WHO) [2]. Considering the years 2019 and 2020, the average, daily, worldwide social media usage by smartphones and internet users was 145 min per day in 2020, which was up from 142 min in the previous year. Currently, the country with the most time spent daily on social media is the Philippines, with online smartphone users spending an average of three hours and fifty-three minutes daily on social media. For comparison, the daily time spent on social media in the United States (US) was just two hours and three minutes.

Currently, the global social network penetration rate is nearly 54%. Western Europe has the highest social media penetration rate at 79%, topping the global social media usage by region; Eastern and middle Africa close the ranking with 10% and 8% usage, respectively [3]. People access social media for a variety of reasons. Users like to find funny or entertaining content and enjoy sharing photos and videos with friends, but they mainly use social media to stay in touch with current events and friends.

Social media profoundly affects smartphone use, interpersonal relationships, and everyday life. According to an online survey conducted in February 2019, social media has improved access to information, ease of communication, and freedom of expression. On the other hand, some respondents also reported that social media had negatively affected their privacy, increased their political polarization, and heightened daily distractions [4]. Social media has such a large global impact on daily life that many internet and smartphone users believe that social media platforms have strongly affected certain aspects of daily life. In this survey, 44% of respondents stated that social media platforms have led to increased political polarization [5,6].

The COVID-19 pandemic has involved the entire world population, putting a strain on national health systems and profoundly changing our lifestyles. Specifically, technology made it possible to carry out essential services required by the population (teaching, communications, public administration, etc.) and support health systems during the pandemic. In this context, wearable devices represent powerful tools for gathering information related to the user’s condition directly from home. These tools were widely employed during the COVID-19 pandemic to avoid the overload of health systems [7,8].

Few studies show a statistical analysis, a model or index relating to the use of SM, the ages and real usage times of users, and how these factors are linked to the onset of pain or pathologies in the neck. Most studies linking neck pain to social media use are statistical questionnaires based on many questions that are addressed to social media users and cross-sectional surveys. A significant study that related SM usage, users’ ages, and neck pain to a certain index or model was presented by H. Yang et al. [9]. This study explored the researcher’s predictive relationship of social media on smartphone use to body esteem in female adolescents and the mechanism underlying these relations. A cognitive–affective framework was investigated, and the relationship between the SM and smartphone use and body esteem was determined by the cognitive internalization of an ideal body image, own aspect comparisons, and social-appearance anxiety. A sample of (*N* = 100) female adolescents aged 13 to 18 was studied. The results were as follows: repetitive SM use tended to cause unhealthy body esteem via intensified cognitive internalization, which triggers appearance comparisons and anxiety regarding negative appearance evaluation. In addition, the time spent in front of the smartphone screen negatively affected the body estimate. This study underestimated the multifactor mechanism that explains the negative impacts of social media and smartphone activities on body esteem in female adolescents who will have low body esteem in the future [10].

An example of neck-pain distribution caused by different factors, including worldwide SM usage, is provided below. A total of 15% of the adult population in Brazil, 4% in China, 20% in the European Union, 5% in Japan, 12% in Russia, and 24% in the US had been diagnosed with a neck pain condition. Moreover, neck pain has an annual prevalence rate exceeding 30% among adults in the US; nearly 50% of individuals will continue to experience chronic neck pain or frequent occurrences. Among adults, between 20% and 70% will experience neck pain that interferes with their daily activities during their lifetime [11,12]. In 1995, the US Department of Defense issued a guideline on the use of computers and tablets in military settings. They recommended that hand-held equipment should not weigh more than 2.3 kg; should be small enough to be held and operated with one hand; and should be no larger than 100 mm high, 255 mm long, and 125 mm wide [13].

Many questions may arise as a result of the reported studies. One of these questions is: Is there any quantitative approach among the subjective-basis approaches to predict muscles’ neck pain associated with SM technology usage? Second, what qualitative basis (criteria) or measurable parameters may enable us to build a new formula or rule to describe or predict the neck pain that may occur? Holding a smartphone device to browse applications and SM at a close distance will cause headaches and eye strain problems. To avoid these health risks and consequences, SM users must use the smartphone device at eye level, change their posture continually, keep an appropriate viewing distance, and reduce the use time of the smartphone device. Some smartphone users do not pay enough attention to their posture while using a smart device.

This research therefore attempts to find an approach or a quantitative method for studying the effect of using SM (i.e., with mini displays) on neck pain and avoiding muscle fatigue with future use. The proposed method uses:

1. The extracted features from X-ray retrospective images using the gray level co-occurrence matrix (GLCM);
2. The two angles measured, and the related area controlled by these angles according to the inclination of the neck vertebrae for normal and abnormal cases (i.e., patients with perceived neck pain);
3. Information about the SM-usage period in hours correlated with the users’ ages. Nine inputs or information were provided to the decision tree (DT) to predict the conditions that may lead to muscle fatigue or produce neck pain;
4. The DT classifier (provided by the Weka package, University of Waikato, New Zealand) can reduce the bias (noise) produced by unbalanced samples. The final phase of this framework is a new, predictive mathematical model utilizing a gene-expression programming (GEP) tool to define the class (normal or abnormal) to which each patient belongs using a MATLAB-based, graphical user interface to support the orthopedic doctor.

### Related Works

The impact of SM usage on neck pain prevalence during the spread of COVID-19 pandemic has been reported in many survey-based studies [14,15,16,17,18].

Access to working at home and distance learning were the most significant factors that imposed neck and shoulder pain. For example, the study reported in Ref. [14] on a sample of 26126 students confirmed neck pain occurrence. In particular, this study aimed to demonstrate the prevalence of neck pain, mainly in medical students, that could be ascribed to electronic device usage during the COVID-19 pandemic. Most students reported a rise in neck aches; the majority noted an increase in neck aches (*n* = 1002—72.3%), whereas *n* = 384 (equal to 26.7%) students indicated the same pain before and during the pandemic. Another work (Ref. [15]) focused on the study of the home use of smartphones in 1045 participants in whom neck-pain symptoms appeared. Demographic and descriptive analyses, a Pearson correlation analysis, reliability tests, and a linear regression analysis were used to process the questionnaire data. The obtained results revealed no correlation between the selected variables; namely, e-learning, online classes, physical fitness, and body pain; and the time spent with smartphones. However, the collected data were affected by personal perception.

In a survey of 2044 Italian students conducted before and after the COVID pandemic, 43.5% of students reported neck pain and 33.5% had lower-back disease [16]. The obtained results demonstrated that musculoskeletal pain in young students could be ascribed to the reduction in physical activity during the COVID-19 lockdown. In a questionnaire study, 129 computer users were asked about functional disability [17]. It was found that 42.9% had neck and upper back pain. In addition, some participants had mild, moderate, and severe functional limitations due to neck and back pain occurrences. This study increased the awareness of having a correct, ergonomic work space. Similarly, in [19], the authors investigated a possible correlation between back pain and COVID-19 infection by analyzing different scientific works presented in the literature. They identified neurological damages induced by disease as a factor of post-COVID-19 chronic pain syndrome. Particularly, the same receptors mediating the access of SARS-CoV-2 into the respiratory systems were present in the neural tissues, thus attributing post-COVID joint pain to neurologic implications due to infection [20].

The alterations to the neurologic system caused by COVID-19 infection are not well understood. However, their effects are evident, such as chronic aches and altered pain sensibilities. Additionally, the extended lockdown periods—actuated to limit the pandemic spread—have exposed the population to risk factors that lead to neck pain mainly due to the prolonged use of electronic devices such as smartphones and tablets [21]. Indeed, the limited display size induces head and neck flexion, causing a postural alteration and resulting in the dysfunction of muscular activity and increasing the cervical load. Several studies have demonstrated a direct correlation between musculoskeletal pain, the number of hours of electronic device usage, and the positive impact of physical exercise on the user’s condition. In particular, the prevalence of neck pain was significantly related to age, sex, health status, job satisfaction, and length of employment. In addition, an increased sedentary lifestyle and incorrect posture appear to promote the onset of musculoskeletal disorders [22]. The main prevention strategies for avoiding these consequences are: reducing smartphone usage time, maintaining correct posture, performing exercise regularly, and teaching healthy habits. In addition to the posture issues, psychological stress is also a cause of neck pain, especially for students and teachers [23,24]. In Ref. [25], the authors investigated the lockdown effect on psychological stress and neck pain (NP). They studied a population of 265 college students by submitting to them a questionnaire related to sociodemographic features, the neck disability index (NDI), self-perceived stress, the visual analogue scale (VAS), and neck pain (NP) frequency. The results demonstrated that 35.6% of students suffered from mild NP impairment during the lockdown, a higher occurrence than before. Students expressed moderate self-perceived stress, whereas 59.3% reported study-related stress. Higher rates of self-perceived, study-related stress, sitting for more than 3 h per day, and a higher VAS score were linked to an increased risk of an NDI ≥ 15 (Nagelkerke’s R^2^ = 0.513, *p* < 0.001).

Similarly, M. Houle et al. analyzed the factors affecting headache and neck pain as well as the effect of wearing a headset on headache and neck-pain intensity in telecommuters over five days [26]. Sixty-two participants were involved, gathering data related to the NBQ (Neck Bournemouth Questionnaire), headache and neck frequency, and wearing a headset (i.e., typology and number of hours). Using a multivariate regression model, the study revealed that only the headache-related disability score was an associated factor of headache occurrence, and only neck-pain-related disability was an associated component of neck-pain occurrence. Headset usage was not linked to increased neck discomfort or headache intensity. In addition, therapists in the United States (US), United Kingdom (UK), and Canada have reported that continuous repetitive and regular usage of a smartphone device for browsing applications and SM may lead to cumulative damage for musculoskeletal and eye users [27]. The studies warned that many static postures of the shoulders, upper spine, and neck could cause physical disorders and diseases later in life. Many optometry and vision-science studies have also shown the same results [20].

Many researchers and scientists suggest some systems and methods that measure the postures of a person and their effects on the neck, shoulder, spine, and eye while using SM on smartphones. For example, S. Arteaga et al. suggested a posture-monitoring system using an accelerometer for stroke survivors [27]. The proposed system consists of a three-axis accelerometer, beeper, light emitting diode (LED), and vibrator and uses many monitoring devices above body parts such as the knees, necks, shoulders, and wrists of subjects. The proposed system warns SM users and stores data when the appropriate posture is detected. In the next study, Farra et al. [13,28] developed a novel system to monitor spine-normal and abnormal positions by measuring the inclination of the SM users’ upper backs and the subsequent stress effect on the spines. Many different sensors have been used to measure this effect, such as an inclinometer and load cells placed around the neck and on the foot of the SM user, identifying a static setting that leads to the lower back pain of the user. Although this model was appropriate for indoor situations such as offices, it could not be applied to the case of SM smartphone users. Another close system of SM monitoring was developed by Baek et al. [29]. They used a two-axis accelerometer to study postures while sitting, standing, and walking. The suggested method divides postures into three groups as a function of the tilt angle between the device and Earth surface.

Some of the researchers in the ergonomics literature studied the effects of different display screens on neck and shoulder pain when compared to a single display. These studies investigated the smartphone screen’s height, viewing angle, and distance from the face [30,31,32,33,34]. Many studies have suggested that a smartphone screen with a lower height and a gaze angle of about 15 degrees is suitable for visual comfort [30,32,33]. However, the most accepted recommendation nowadays is to position the smartphone screen at eye level. In other published studies, positioning the display screen of a personal computer (PC) or a smartphone in a central position was compared with placing the screen at a position angled to the left and to the right [31]. It was found that the angled position was associated with a significant increase in neck and shoulder muscle activity and head movements. Finally, Straker et al. studied the flexion angle differences between adolescent computer users and non-computer users while using social media. They found that computer users demonstrated increased neck flexion and pelvic tilt [35]. They also showed that more time spent on the computer while using social media was associated with an increased bending of the head and neck, especially in boys, and of the lower back in girls (leading to lumbar lordosis) [36].

This manuscript is organized as follows: Section 1 contains the introduction to SM usage. Section 2 presents the method and materials, including the results of the decision-tree classifier and statistical analysis. In Section 4, the discussion of the obtained results is conducted. The final section is the conclusion.

## 2. Materials and Methods

### 2.1. Data Collection and Study Design

Based on retrospective X-ray neck images, a cross-sectional study was conducted on 46 patients with neck-pain occurrences in the Arab Medical Centre (Amman, Jordan). The data collection took place between 15 February 2020, and 18 April 2020, during which 46 X-ray neck images were captured using an X-ray imaging machine (i.e., the Definium Tempo^TM^ fixed X-ray system (GE HealthCare, Chicago, IL, USA)), with a matrix size of 2000 × 2500. For a 35 cm × 43 cm cassette, this digital matrix corresponds to a pixel size of 175 μm and a limiting resolution of three line pairs per millimeter (namely, a spatial resolution of 0.1 mm). First, participants completed a short survey before undergoing the orthopedic examination.

Table 1 briefly illustrates the survey content provided to the patients; the age, gender, duration of time spent using SM devices (in hours), presence of chronic pathologies such as diabetes or high blood pressure, and the pain type for which the orthopedic visit was necessary were the information requested from the participants.

**Figure 1:**
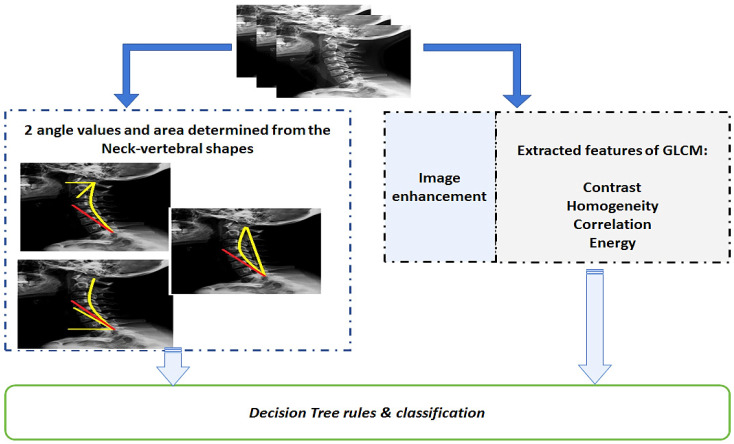
Block diagram of the proposed system for predicting the conditions leading to neck pain (GLCM: gray level co-occurrence matrix).

X-ray neck images were acquired under the supervision of an orthopedic doctor, with patients seated on chairs in front of the X-ray machine. Patients with many healthcare problems, such as blood pressure and diabetes, were also considered in the research study. The orthopedic doctor questioned all patients about the time duration of their use of SM devices. Prior to visiting the doctor, patients thought they had ear pain, not neck-muscle tension; therefore, the X-ray imaging was essential. The proposed approach relating to the image-processing and subsequent extraction of GLCM parameters in order to determine the neck-pain pathology due to the intensive use of SM devices is illustrated in Figure 1.

### 2.2. Ethical Approval

This research study was approved by the Institutional Review Board (IRB) of the Hashemite University (No. 26/7/2021/2022). Informed consent was obtained from patients; it was reported on the first page of the questionnaire submitted to them, written in both Arabic and English. It explained the study’s aims and emphasized the confidentiality of given information. The participants were able to withdraw from the study at any point. No identifying information was obtained through the study, and all data collected were solely used for the statistical analysis.

### 2.3. Inclusion and Exclusion Criteria

The research study was conducted on 46 patients in the Arab Medical Centre (Amman, Jordan). All the participants declared in the completed questionnaires (Table 1) that they used SM devices or smartphones intensively every day. Patients with any long-term pathologies, musculoskeletal diseases or anomalies, or those who had previous surgeries on the neck or shoulders were not included in our study.

Table 2 reports the pathology types or painful parts of the body, as declared by the patients in the questionnaires, and the number of patients for each pathology. The demographics of the patients who participated in the study are presented in Table 3. The number of female patients was twenty-seven, with a mean age ± STDev (standard deviation) equal to 33.70 ± 6.82, respectively, and the number of male patients was nineteen, with a mean age ± STDev equal to 41.42 ± 8.10, respectively.

As can be observed, imbalanced datasets are a regular occurrence in healthcare applications. In this work, the class ratio between the majority (38 abnormal) and minority (8 normal) samples is only five to one and does not represent a significant imbalance. The machine learning literature on this problem has given three primary solution possibilities:

(i) To restore balance in the training set and avoid the creation of bias in the first place, it is possible to under-sample the large class or to over-sample the small class [37];
(ii) Alternately, one can change the costs (shift matrix) associated with misclassification in order to prevent any bias [37];
(iii) A further precaution is to replace precision with so-called balanced accuracy [37].

In this study, the first and second solutions were evaluated by choosing new parameters for the “weka.classifiers.trees.J48” package (from the University of Waikato, Hamilton, New Zealand) that was applied to our dataset [38]. The dataset resampling procedure was implemented as follows: in the Weka software version 2022 [38], regarding the oversampling feature (to increase the minority group), we used the function “classifiers.meta.CostSensitiveClassifier” in default mode, which reweights the training instances to take a given misclassification cost matrix into account and then uses the classifier built from the reweighted data. The weighted average of the receiver operating characteristic (ROC) area and the F-measure (provided later in Section 3.2) demonstrate that the results obtained after the oversampling procedure are extremely close to the previous resampling.

### 2.4. Angles and Area Measurement

**Figure 2:**
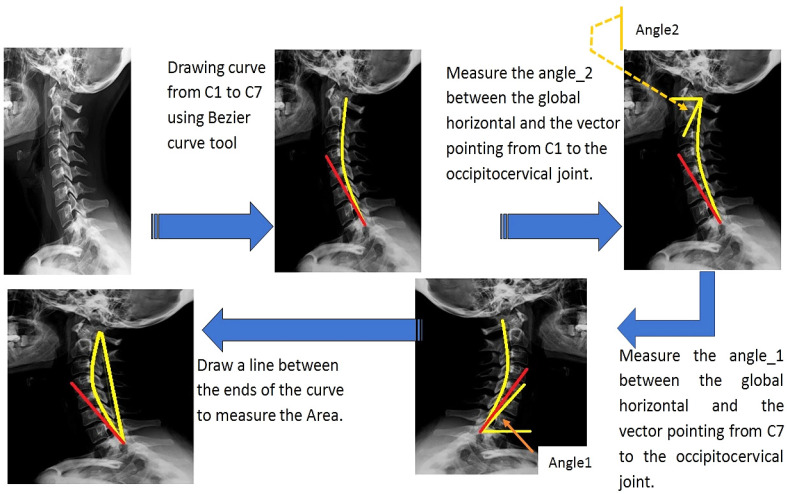
Angles and area measurements used as input of the decision tree (DT) to predict neck pain.

Often, the practical approach to distinguishing between normal neck conditions and pathological cases due to the use of SM devices is to measure only one angle (i.e., angle_1, the angle between the global horizontal and the vector pointing from the C7 vertebra to the occipito-cervical joint). Instead, in this study, two angles were measured; angle_1 and angle_2 (namely, the angle between the global horizontal and the vector pointing from C1 vertebra to the occipito-cervical joint), together with the area between them. The procedure of measuring angles and the area between them is shown in Figure 2. The X-ray images showed that a curve can be drawn through the neck vertebrates, from C1 to C7 vertebrae, using the Bezier curve tool. Next, the angle between the horizontal line and the curve at the C1 vertebra was measured using the angle tool (named angle_2, as shown in Figure 2). The angle_1 was detected using the angle tool between the horizontal line and the curve at C7 vertebra (as shown in Figure 2). Finally, the area between the two angles was measured by drawing the line between these ends (the C1 and C7 vertebrae). All parameters, including angle_1, angle_2, and area, were measured for all patients with pathological (declared pain) and normal conditions (no declared pain). With the use of the image analysis software Image J version 1.801 (NIH, Bethesda, MD, US), the angles and area parameters were determined.

### 2.5. Features Extraction from the Gray Level Co-Occurrence Matrix

The X-ray images of the human neck with or without muscle tension or painful pathology have the same specifications; however, they are different from the X-ray images of an abnormal lung affected by coronavirus or with the presence of tumor lesions. The X-ray images of lungs can be easily applied to deep-learning machine algorithms to predict abnormalities. In the case of X-ray images of the neck, there are no tumors or infections to be detected by the machine-learning algorithm; thus, finding specific statistical features through an image-based analysis is very important. The attempt to use the GLCM method may be a suitable solution.

The parameters that can be extracted by means of the GLCM method depend on the image quality that may be accordingly correlated with the neck shapes. GLCM was applied to the X-ray images to extract four additional parameters [39,40], which are valuable for evaluating neck muscle pain. The GLCM functions characterize an image texture by calculating how often pairs of pixels with specific values and specified spatial relationships occur in an image, creating a GLCM, and then extracting the statistical measures from this matrix. The GLCM method calculates how often a pixel with the intensity (gray level) value *i* occurs in a specific spatial relationship to a pixel with the value *j* (cell value). The GLCM parameters extracted in this research work are:

(i) Contrast: the separation between the brightest and darkest image area; namely, the difference between the highest and lowest values of the adjacent set of pixels.

(1) $$contrast=\sum_{i,j=0}^{N-1}P_{i,j}{\left(i-j\right)}^{2}$$

(2) $$P_{i,j}=\frac{V_{i,j}}{\sum_{i,j=0}^{N-1}V_{i,j}}$$

where *N* stands for the number of gray levels and *P*(*i*,*j*) is the normalized value of the gray-scale at positions *i* and *j* of the kernel with a sum equal to 1. *V* is the value in the cell *i*,*j* of the image window; if *i* and *j* are equal (*i* − *j*) = 0, the cell is on the diagonal.

(ii) Homogeneity: the closeness of the distribution of elements in the GLCM to the GLCM diagonal. It is defined as:

(3) $$homogeneity=\frac{P_{i,j}}{1+{\left(i-j\right)}^{2}\;}$$

In general, the cell or pixel value attribute can have a variety of display formats, including empty, numeric, text, Boolean, or error; in this case, it is at a a gray-scale intensity level. Windowing is a certain size of resolution with a cell format that can be applied to the original image in the form of gray-level mapping, contrast stretching, histogram modification, or contrast enhancement such as a filter, mask, or kernel, etc. [41].

(iii) Correlation: the linear dependency of gray levels on those of neighboring pixels. This indicates that there is a predictable and linear relationship between two neighboring pixels within the window, expressed by the regression equation. The correlation can be represented mathematically as:

(4) $$correlation=\sum_{i,j=0}^{N-1}P_{i,j}\left[\frac{\left(i-{µ}_{i}\right)\left(j-{µ}_{j}\right)}{\sqrt{\left(\sigma_{i}^{2}\right)\left(\sigma_{j}^{2}\right)}}\right]\;$$

where μ and σ are the GLCM mean and variance, respectively.

(iv) Energy: computed as the square root of an angular second moment:

(5) $$Energy=\sum_{i,j=0}^{N-1}P_{i,j}^{2}$$

**Figure 3:**
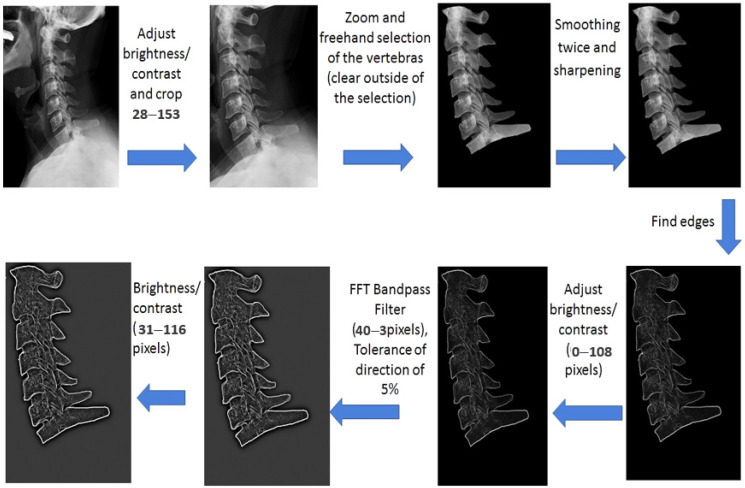
Features extraction using the GLCM method applied to the neck’s X-ray images.

For pre-GLCM employment, the images need to be enhanced or pre-processed. The pre-processing steps aim to remove the background, eliminate the noise, and to visualize the edges of the vertebrae (bones), as is shown in Figure 3. The overall flow of image pre-processing implemented in this research work is reported below.

(1) Adjusting the contrast and brightness of the image; cropping to specify the position of the neck;
(2) Magnification of the image and freehand selection of the vertebrae;
(3) Smoothing many times and improving the image sharpness;
(4) Finding the edges of the vertebrae using the image edge detection method;
(5) Adjusting the brightness and contrast of the image (0–108 pixels);
(6) Applying the fast Fourier transform (FFT) bandpass filter to remove low and high spatial frequencies responsible for image blurring [42]. This filter has been designed to smooth variations of the X-ray image (bright or dark patches) with sizes larger than 40 pixels and to strongly attenuate insignificant spots smaller than 3 pixels. Note that these values are half the spatial frequencies of the actual cutoff frequency. The cutoff frequency is very soft, so the bandpass filter will also significantly attenuate the spatial frequencies in the center of the bandpass unless the difference between the two values is greater than a factor of five or so. It can also suppress the horizontal or vertical stripes created by scanning an image line by line with a direction tolerance of 5%;
(7) Further adjusting to the brightness and contrast of the image;
(8) Resizing the image size to 256 × 256 pixels and completing a gray-scale conversion;
(9) Applying the GLCM method using Matlab software (version. R2022a, MathWorks, Inc., Natick, MA, US) to extract contrast, homogeneity, correlation, and energy parameters.

### 2.6. Descriptive Variables

The time duration of the neck-pain-causing use of SM devices in hours (H.mean) was categorized into five groups, with a width of each group up to 4 h. These groups were: (1) no perceived pain (normal subject); (2) pain after 2–3 h; (3) pain after 3–6 h; (4) perceived pain after 6–9 h; and (5) perceived pain after more than 9 h. The descriptive variables used in this study were the age, H.mean, angle_1, angle_2, area, contrast, homogeneity, correlation, and energy. These are presented in Table 4, where statistics contain the mean, standard deviation (STDeviation), minimum (Min), maximum (Max), and standard error (Std. Error) values. The standard error provides the precision of a sample mean by accounting for sample-to-sample variability; it represents the predicted measurement error in a single individual’s score. The used formula to calculate the Std. Error is $STDeviation/\sqrt{n}$, where *n* is the number of samples [43].

It is important to point out that the deterministic data reported in Table 4 related to age, H.mean, angle_1, angle_2, area, contrast, homogeneity, correlation, and energy have no random components. The same input values generate the same output. As a result, the standard-error values from statistical data models do not have their usual meanings. A high statistical significance (namely, a low standard-error value) is an indication of an effect due to a model term. However, it is not possible to construct valid confidence intervals for effects or model predictions.

### 2.7. Decision Trees

Decision trees (DTs) are standard machine-learning methods used in classification [44]. The DTs are usually known for their simplicity, reliability, and classification performance [45]. They have been used for a variety of biomedical applications. The DT’s generation depends on performing entropy [46] and information gain (IG) calculations [47]. The DT algorithm named C4.5, introduced by Quinlan [48], was used as a data classifier in this work known as the “weka.classifiers.trees.J48” package (developed by the University of Waikato, Hamilton, New Zealand). The entropy calculations were performed to determine the information gain as described by Shannon in the following equation [49]:

(6) $$E=-\sum_{i=1}^{n}p_{i}\log_{b}p_{i}\;$$

The information gain calculations include the comparison between the entropy of each attribute with the total entropy of the dataset. These calculations are performed on each node of the decision tree, as is illustrated and discussed below in Section 3.2. The attribute is selected for that node according to the highest information gain calculation. The calculations are then repeated recursively for each branch of the DT.

### 2.8. Mathematical Predictive Model for the Neck Pain Diagnosis

**Figure 4:**
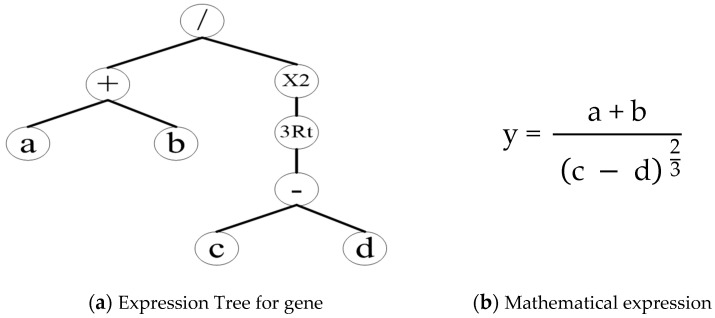

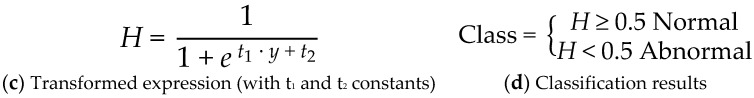
An example of the GEP model: (**a**) expression tree (ET), (**b**) the matching mathematical expression, (**c**) the transformed expression (H) using the sigmoid function, and (**d**) classification limits and categories. Note: a, b, c, and d are the feature variables of the processed model, while t_1_ and t_2_ are constants of the sigmoid function for developing the transformed expression (the related values after having implemented the mathematical model to the specific case are specified in Equation (9)).

A mathematical, predictive model with a MATLAB graphic interface has been developed to help the orthopedic doctor predict the onset of neck pain. The gene expression programming (GEP) technique has been used to create the model [50]; the genes are made up of numerous linked chromosomes that look like trees and have several variables structured in a start and end point. These trees are known as GEP expression trees and can be expressed mathematically; an example is shown in Figure 4.

The GEP method involves several mechanisms, including building and expressing chromosomes for the dataset, running the framework, evaluating fitness criteria such as the root-mean-square error (RMSE) or the coefficient of determination (R-squared), and terminating the programming process if the fitness requirements are met. Otherwise, the gene expression must be replicated through variable crossover or mutation. Figure 4 depicts a GEP algorithm as an expression tree (ET). The algorithm starts by implementing the GEP process, which comprises the creation of the chromosomes with the required function settings and fitness criteria for the variables a, b, c, and d (Figure 4a). The chromosomes are then executed and transformed into the tree expressions (TEs). The framework processing terminates if the TEs meet the fitness criteria. However, if the fitness criteria are not met, the variables are altered by performing one or more genetic operations (crossover or mutation) on selected TEs. The TEs are eventually transformed into mathematical expressions to make them more convenient for design applications.

In this work, the experimental database was processed with GeneXproTools 5.0 software (developed by Gepsoft Lda, Capelo, Portugal) to create the GEP model for class prediction (normal or abnormal). The database was separated into training and testing categories to ensure the generalization and validity of the constructed model across a wide range of variables and to minimize overfitting. The training data were only utilized to create the GEP model, whereas the testing data were used to test the model validity and accuracy. In this study, 31 points (67% compared to the total of 46 patients participating in the study) were selected at random for the training data category, whereas 15 points (equal to 33%) were used for the testing data category.

Several GEP process settings are critical to developing the most efficient model. Table 5 summarizes the GEP settings used in this study. The data population consisted of 46 individuals and the fitness criteria are based on the least receiver-operating characteristic (ROC) value. There were ten independent variables: gender, age, time spent on social media (H.mean), angle_1, angle_2, area, contrast, homogeneity, correlation, and energy. Table 5 displays the parameters chosen for the GEP algorithm; there are five genes with a maximum tree depth of eight levels, and the three trees are linked together by multiplication.

**Figure 5:**
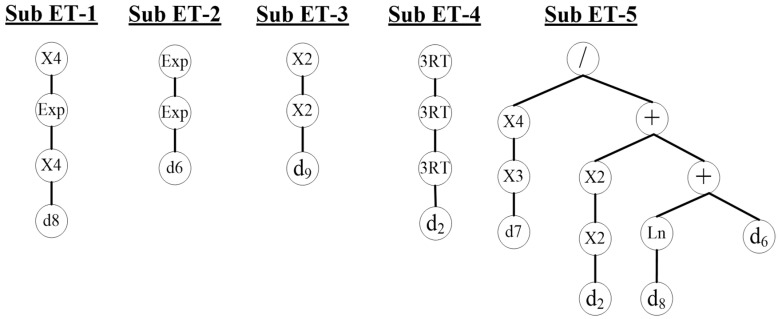
GEP model in the ET form.

Figure 5 depicts the developed GEP model in the ET form, consisting of five sub-trees (sub-ET1, 2, 3, 4, and 5) linked by multiplication (Equation (7)). Out of the total of ten parameters reported in Table 6, only six variables (i.e., *d*_0_, *d*_2_, *d*_6_–*d*_9_) were employed in the developed mathematical model, chosen by the fitness function ROC. If the fitness conditions were not met, the variables were modified by performing one or more genetic operations on the selected TEs (crossover or mutation).

The product of the five sub-trees yielded the predicted classification. The GEP model represented in Figure 5 as an ET was transformed into the mathematical expression of Equation (7). After further simplification, Equation (9) was obtained, relating to the H-parameter that establishes which class the patient belongs to. The values of the constants t1 and t2 (shown in the general expression of the parameter H in Figure 4c) were obtained from the GEP model and were equal to −1797.29 and 7.28, respectively. As is reported in Figure 4d, if H assumes values greater than or equal to 0.5, it is a normal condition (namely, no perceived pain). If H is less than 0.5, it belongs to the abnormal class, which means the presence of pain perceived by the user.

Equation (7) (i.e., *y*) represents the GEP mathematical model, in which each mathematical term corresponds to an expression tree (ET). The multiplication function connects these terms. The highlighted parameters taken from the X-images and optimized based on the ROC fitness function are the variables *d*_2_, and *d*_6_–*d*_9_. In Table 6 these variables have been defined.

(7) $$y={\left(e^{d_{8}{}^{4}}\right)}^{4}\times e^{e^{d_{6}}}\times d_{9}{}^{4}\times d_{2}{}^{\frac{1}{27}}\times \frac{d_{7}{}^{\frac{1}{12}}}{d_{2}{}^{4}+Ln\left(d_{8}\right)+d_{6}}\;$$

(8) $$y={\left(e^{Correlation^{4}}\right)}^{4}\times e^{e^{Contrast}}\times Energy^{4}\times Time^{\frac{1}{27}}\times \frac{Homogeneity^{\frac{1}{12}}}{Time^{4}+Ln\left(Correlation\right)+Contrast}\;$$

(9) $$H=\frac{1}{1+e^{-1797.29\times y+7.28}}$$

The term *H* is the transformed expression using the sigmoid function [50]; its numerical limit and related class were determined as is shown in Figure 4d.

## 3. Results

This research aimed to define a quantitative approach for predicting conditions that lead to neck pain resulting from the intensive use of SM devices by considering the duration of their use, in hours, as a variable. The proposed method, presented in Figure 1, is able to find the appropriate parameters for predicting the SM effects. Figure 2 shows the first step of the proposed approach of finding the appropriate angles and the area between them associated with the variability of SM-device usage. With the advantages of the Bezier curve tool, the Image J version 1.801 software (NIH, Bethesda, MD, U.S.A.) was employed to draw the line via the vertebrae and then draw the tangent line at the level of the C1 and C7 vertebrae. Maintaining an accurate performance during the measurement is essential because the difference between the condition of no perceived pain (normal) and that of perceived pain (abnormal condition) is very sensitive.

Figure 3 shows the second part of the SM parameters detection; namely, the steps of image quality enhancement before extracting the features (parameters) of contrast, homogeneity, correlation, and energy through the GLCM method. All the steps in Figure 3 were performed to visualize the vertebrae edges that reflect the quality of the features. The statistical description for all the obtained parameters is presented in Table 4 (Section 2.6).

### 3.1. Results of Statistical Validation for the Objectively Calculated Parameters

The Statistical Product and Service Solutions (SPSS) statistics software, version 21.0 (developed by IBM Software Group, Chicago, IL, USA), was used to analyze the data. A one-way ANOVA test and an independent-sample *t*-test were used to analyze the differences in age, use duration, neck-pain duration, and GLCM features. Some studies used both multiple linear regression and binary logistic regression analyses [51,52,53]. In this work, however, the same testing protocol used in other works [49,54,55] was applied, but with a new machine-learning model known as the decision tree. The decision tree can predict the occurrence of neck pain better than logistic regression based on the calculated entropy and the *if–then* rules. The redeployment of machine learning and genetic expression as predictive models will allow for improved rapid diagnostic tools in this field. The obtained progress may end with developing an in-cloud application in which physicians scan the X-ray image while entering the patient’s gender and age to predict the neck condition, especially for those with excessive SM-device usage.

**Figure 6:**
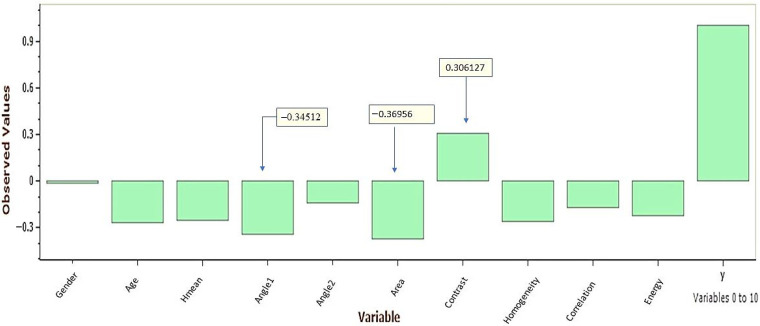
The best correlation coefficient vs. the response (perceived neck pain/no perceived neck pain) to angle_1, area, and contrast.

**Figure 7:**
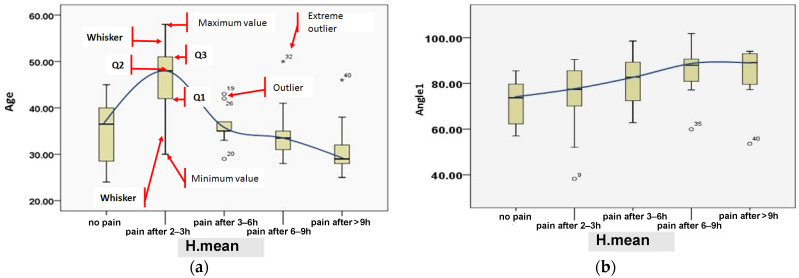

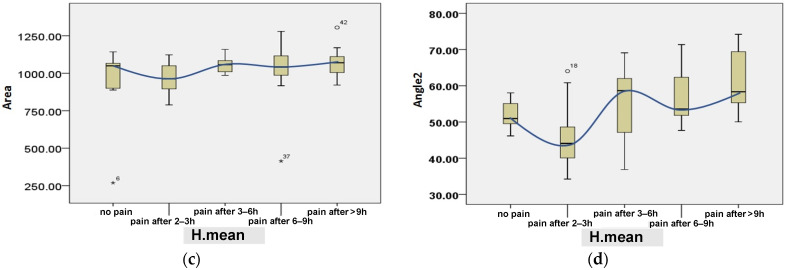
Effect of SM usage examined by time duration (i.e., H.mean) on age (**a**), angle_1 (**b**), area (**c**), and angle_2 (**d**). In Figure 7a for the variable age, the following values are reported: the first quartile Q1, that is the value below which 25% of the values in the data set are found, the median of the data values Q2, the third quartile Q3, that is the value below which 75% of the values in the data set are found, the interquartile range (IQR) = (Q3 − Q1) namely the middle 50% of the data set. The lower whisker is a line from Q1 to the smallest data point within the (1.5 × IQR) range from Q1. The upper whisker is a line from the Q3 to the largest data point within the (1.5 × IQR) range from Q3. The mild outliers, values smaller than [Q1 − 1.5(IQR)] or larger than [Q3 + 1.5(IQR)], represented by circles; the extreme outliers, values smaller than [Q1 − 3(IQR)] or larger than [Q3 + 3(IQR)], represented by stars. Outliers in data, regardless of whether they are legitimate or the consequence of errors, are generally worthy of investigation. Figure 7a’s upper outliers, ages 19 and 26, indicate whether or not these patients, ages 19 and 26, had worked 3–6 h using SM devices. Instead, the patient being observed (*32) is an error, as it does not fall within the range of 6–9 h and is eliminated.

**Figure 8:**
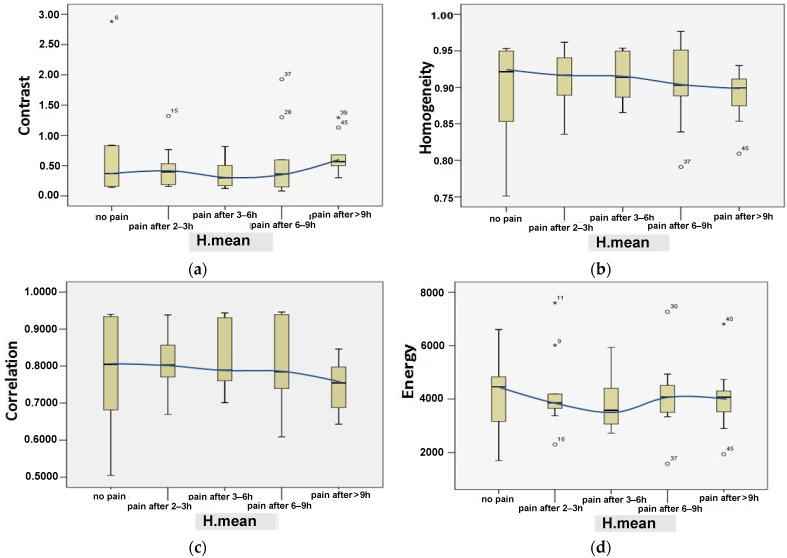
The effect of SM usage examined by time duration (i.e., H.mean) on the contrast (**a**), homogeneity (**b**), correlation (**c**), and energy (**d**). Mild outliers, the values smaller than [Q1 – 1.5(IQR)] or larger than [Q3 + 1.5(IQR)], represented by circles; the extreme outliers, values smaller than [Q1 – 3(IQR)] or larger than [Q3 + 3(IQR)], represented by stars, as shown and detailed in Figure 7a.

Table 7 demonstrates the correlation between all parameters (−1≤r≤+1) and the response to neck condition (i.e., no perceived pain-normal condition or perceived pain-abnormal condition). In contrast, the graphical representation for correlation vs. response is shown in Figure 6. In Figure 7 and Figure 8, a comprehensive analysis was performed, taking into account all parameters: age, angle_1, angle_2, area, contrast, homogeneity, correlation, and energy by H.mean, utilizing the box-plot, while the central value was 95%.

### 3.2. Results of Using the Decision Tree Method

**Figure 9:**
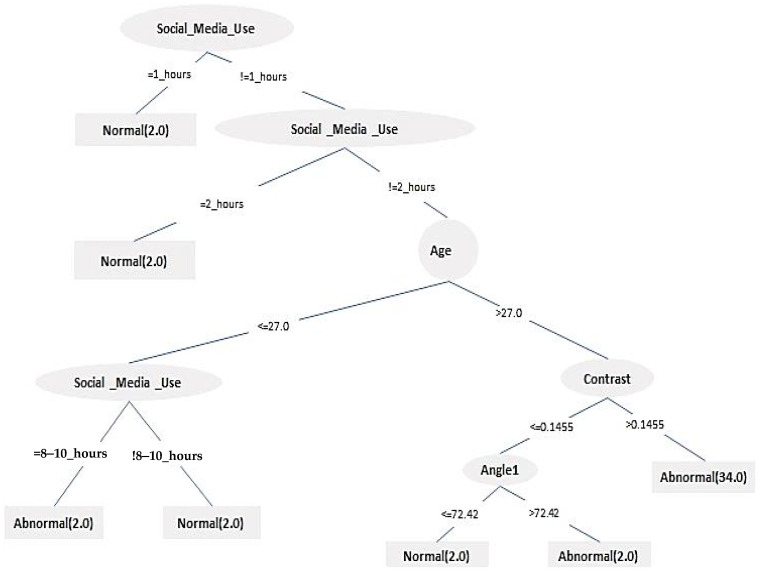
Generated decision tree to predict the effect of SM usage on neck pain.

The dataset has eleven attributes and two classes. These attributes are gender, age, Social_Media_Use (H.mean), angle_1, angle_2, area, contrast, homogeneity, correlation, energy, and diagnosis. The attribute “diagnosis” formed the two classes. Figure 9 shows an unpruned DT built using 46 instances; two-thirds (66.7%) of the dataset were used to build the DT, and one-third (33.3%) of the dataset was used for testing purposes to assess the performance of the learning approach (16 samples). The size of the DT generated was thirteen nodes with seven leaves in total, as is shown in Figure 9.

In this study, the confusion matrix of the test dataset was maintained by combining normal and abnormal patients (34%; 16 samples). The next phase involved reducing the disparity (unbalanced ratio) between abnormal and normal samples. Thus, the DT classifier’s new function, the cost matrix, was activated with entries weighted [(0, 1); (5, 0)] (brought by the Weka package). Consequently, the relative costs of misclassifying true positives and true negatives were calculated. Note that the steps provided earlier had an effect on the confusion matrix that resulted in the following prediction parameters: *TP* = 13, *TN* = 2, *FN* = 1, and *FP* = 0 (as are reported in Table 8).

The results show that 13 out of 16 instances were classified correctly, with an accuracy of around 94%. Furthermore, a good accuracy of class abnormal (perceived neck pain) was obtained with an F1-score equal to 0.95. The results of the DT performance (shown in Table 8) have been calculated using Equations (10)–(13):

(10) $$Precision=\frac{TP}{\left(TP+FP\right)}$$

(11) $$Recall=\frac{TP}{\left(TP+FN\right)}$$

(12) $$F1-Score=\frac{2\times Precision\times Recall}{Precision+Recall}$$

(13) $$Accuracy=\frac{TP+TN}{TP+TN+FP+FN}$$

*TP* stands for true positive, *FP* for false positive, *TN* for true negative, and *FN* for false negative. The expression “perceived neck pain” refers to abnormal samples; “perceived no pain” refers to normal cases. The confusion matrix reflects these terms as follows:

− *TP*: true positive represents a case or patient with neck pain (abnormal) detected correctly;
− *FP*: false positive represents a case or patient without neck pain (normal) detected as abnormal (perceived neck pain);
− *TN*: true negative represents a case or patient without neck pain (normal) detected correctly;
− *FN*: false negative represents a case or patient with neck pain (abnormal) detected as normal (no perceived neck pain).

In addition, the 9-fold cross-validation setup was applied during the run of the new “CostSensitiveClassifier” settings of the DT, resulting in the successfully classified instance and the weighted F-score being 93.75% and 0.94, respectively.

Noteworthily, the selection of the DT to be applied to this dataset was a successful choice because, while the DT algorithm is suitable for large and small datasets, it may not be suitable for large-scale metadata. If the dataset is too large, the algorithm may become too complex, leading to an overfitting issue (a major risk). In this work, the training–testing split protocol was selected as 70%–30% or approximately 30 samples for training vs. 16 samples for testing. On the other hand, the dataset is unbalanced—i.e., with 38 (corresponding to 82.61%) abnormal cases and 8 (corresponding to 17.39%) normal ones (Table 3)—which facilitates decision-making. Information gain (IG) helps measure the uncertainty reduction of a certain feature. It also helps to decide which feature is good as a root node. Therefore, detecting 13 (TP) out of 16 samples during the test algorithm validation indicates very efficient decision-making. In the DT, the wrong decision is usually related to the last leaf in one of the branches; it can be avoided if the DT is trained with a larger dataset. Overall, the DT technique is considered one of the easiest techniques in machine learning, with the capability of being applied even in real-time situations where speed and simplicity are an advantage.

The DT shown in Figure 9 indicates that the more time is spent on social media, the more abnormality and neck pain occurs. A set of classification rules generated by the DT are summarized in Table 9.

### 3.3. Results from the Developed Graphical GEP Predictive Model

**Figure 10:**
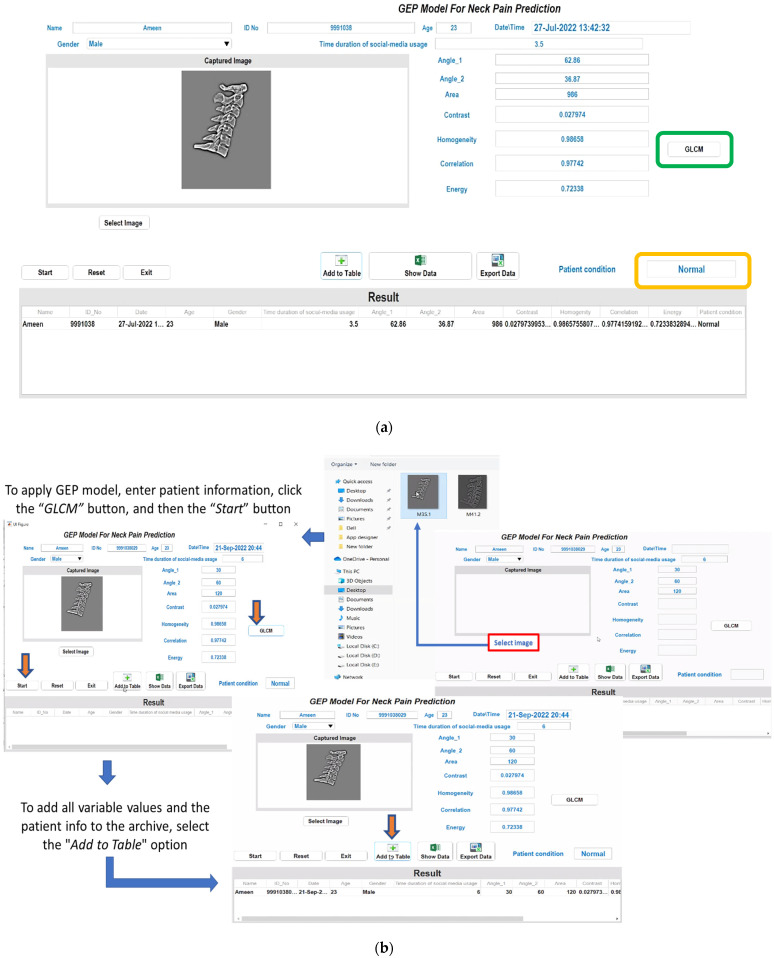
Screenshot of the MATLAB-based GUI of the developed predictive mathematical model (**a**); the operation procedure of the MATLAB application with indicated the various phases that follow one another (**b**).

The MATLAB graphical user interface (GUI) for the developed mathematical prediction model based on Equations (7)–(9) is shown in Figure 10a. The MATLAB GUI’s functionality begins with the selection, directly by the common user or by the orthopedic doctor, of the pre-processed X-ray neck image (by the *Select Image* button) from the specific folder. After that, the user must enter the *d*_3_–*d*_5_ geometrical parameters’ values (i.e., angle_1, angle_2, and area) previously obtained from the orthopedic doctor by viewing the X-ray image with a specific software. Thereafter, by clicking the “GLCM” button on the left of Figure 10a (green box), the *d*_6_–*d*_9_ parameters are automatically determined using the GLCM function (variables defined in Table 6). The geometrical and GLCM data are then combined with additional information, such as the user’s gender, age, and time duration of SM usage (H.mean) to predict the patient condition (i.e., no perceived neck pain—normal condition or perceived pain—abnormal condition) by the obtained H-value. The predicted class will be displayed on the GUI as “Normal” (if the H parameter value is greater than 0.5) or “Abnormal;” that is, perceived pain from the patient for H values less than 0.5 (orange box in Figure 10). The GUI also features an expanded function for archiving the patient information by transferring all the data in an excel sheet for later use (storage and printing). For further clarity, Figure 10b illustrates the different steps of the MATLAB application, as are described above. An MP4 video illustrating the various operating steps of the developed MATLAB application is available to the reader as Supplementary Materials.

Table 10 presents the results of testing the prediction GEP model (described in Section 2.8) on 46 real patients (the total number of participants to the research work, numbered in Table 10). All the X-ray images were saved in the specific folder and named with the same identification number, to be selected (by the “Select Image” button) and then processed. The normal class (that is, no perceived pain corresponding to H values greater than 0.5) is indicated with value 1; instead, the zero indicates belonging to the abnormal class (that is, perceived pain from the patient for H values less than 0.5). After the model validation/testing, the obtained accuracy calculated by Equation (13) was 91.30%, in which:

− *TP* = 8, when the patient condition (as reported in the questionnaire) is normal (i.e., no perceived pain, value 1) and the predicted class is normal (namely, H value ≥ 0.5);
− *TN* = 34, when the patient condition (as reported in the questionnaire) is abnormal (i.e., perceived neck pain) and the predicted class is also abnormal (i.e., H value < 0.5);
− *FP* = 4, when the patient condition (as reported in the questionnaire) is abnormal (i.e., perceived neck pain) and the predicted class (by the GEP prediction model) is normal (namely, H value ≥ 0.5);
− *FN* = 0; when the patient condition (as reported in the questionnaire) is normal (i.e., no perceived pain, value 1) and the predicted class is abnormal (H value < 0.5).

The trained model learns from the training database repeatedly until the predicted neck pain achieves performance criteria such as RMSE, R-squared, or ROC. Based on the ROC, the variables enrolled in the GEP mathematical model are *d*_0_, *d*_2_, and *d*_6_–*d*_9_. If the parameters (variables) with a proportional relationship do not meet the fitness function, they are not considered. For this reason, other parameters such as d1, *d*_3_, *d*_4_, and *d*_5_ were left out of the model (namely, they are not present in Equation (7)). The obtained testing accuracy equal to 91.30% is less than that of the DT (equal to 94%, as reported in Table 8). This is supported by the different fitness functions; the entropy function was utilized in DT, while the ROC function was utilized in the ETs. The four errors present in Table 10 (“Wrong FP” in class matching), corresponding to 8.69%, indicate that the abnormal condition’s variables (parameters) were incorrectly predicted, and thus the resulting H value (>0.5) led to the incorrect classification (as normal).

### 3.4. Further Comorbidities

Comorbidities are the association of several diseases and health problems that can lead to the onset of a progressive or chronic disease. The co-occurrence of new diseases might disappear and occasionally progress. However, the engagement and reliance on SM entertainment and smartphones has become a kind of obsessive lifestyle over the last decade. Researchers have attempted to investigate this contentious fact from a new perspective. For example, 159 adults from Midwestern University took part in a research study on smartphone dependency (nomophobia) [56]. It was discovered that using a phone while driving and being female are strong positive indicators of smartphone dependency.

Much research, such as the current study, have suggested that patients using SM devices adopt forward postures. At the same time, web browsing, video-game playing, emailing, and texting are linked to a variety of health issues including kyphosis, which is associated with pulmonary and cardiovascular disorders. Furthermore, new research indicates that when patients stare down at a smartphone, personal computer, or tablet, they lower their heads and rotate their shoulders while looking down. As a result, rib muscular limitation or tightness, as well as inappropriate posture during movement, make normal breathing difficult [57]. Neck flexion and weight have a substantial proportional relationship, which is most likely owing to decreased physical activity [36,58]. Headaches, such as migraines, have been examined in school-age children and adolescents, and they vary by age and gender [59]. One study discovered a 48% prevalence of headaches among school-aged children aged 7 to 18 years, while another discovered that headaches are the most prevalent discomfort among schoolgirls (42%) [60].

## 4. Discussion

This work is a new attempt to study the effects of social media entertainment usage that may lead to serious neck pain. Patients complained of neck pain during their visit to the orthopedic doctor before undergoing X-ray radiography; at the same time, they reported through the questionnaires that they used SM devices for a significant number of hours (H.mean). To develop a reliable approach to assessing SM impacts on the neck muscles, a quantitative method was performed on 46 retrospective, neck-vertebral X-ray images. Thus, the studied parameters were detected based on two syncretized steps: (1) three parameters, angle_1, angle_2, and the area between them, were measured by drawing a Bezier curve through the vertebral neck path; (2) the second step relayed the image quality in which the GLCM was applied to extract four features of contrast, homogeneity, correlation, and energy. The progress of the neck-muscle fatigue was classified as normal (no pain) and abnormal (pain), and all parameters were examined by the time duration of SM usage known as H.mean.

Table 7 shows the correlation between all parameters and the response, whether normal (no perceived pain) or abnormal (perceived pain) cases. The absolute correlation changes from −1 to +1 (i.e., −1 ≤ *r* ≤ +1) were considered, while in Figure 6, the best correlation was calculated for the angle_1, area, and contrast of −0.34512, −0.36956, and 0.306127, respectively. This result was proven in Figure 7b, in which the value of angle_1 in normal subjects is the smallest if the user interacted with SM devices for an average of only two hours. It is worth noting that the greater the interaction with SM devices, the greater the increase in angle_1. In other words, dealing with SM devices for more than 2 h may lead to neck muscle tension. In correlation with the patient age (Figure 7a), it is obvious that candidates aged less than 30 years were qualified to develop neck pain if they interacted with SM for more than 6 h.

Concerning angle_2, as is shown in Figure 7d, a slight increase, with times ranging from 3 to 9 h, is observed. Also, notably, the area is not proportional to the angles increasing, which means that area is affected (becoming smaller) by the angles increasing in the time interval of 3–9 h and above, according to Figure 7c. Image quality may be affected by fatigue and stress levels, as is shown by the GLCM features in Figure 8. For example, a significant difference was observed related to the images’ contrast for normal (no perceived pain) and pain conditions after more than 6 h usage, as shown in Figure 8a. The energy parameter clearly differentiates between no pain and suffering claims. Alternatively, the average values for the energy indicator remain constant with a temporal correlation (2–9 h), indicating that neck pain was unaffected, as is shown in Figure 8d. However, given that the change rate for these values for a time between 3 and 9 h is similar, it appears that the difference between no pain and discomfort in terms of correlation and homogeneity cannot be counted. (Figure 8c,d).

In this study, the DT classifier was employed successfully. Understanding and predicting the neck pain caused by SM devices can be modelled using the DT classifier’s advantages over other classifiers. These if–then rules are presented in Table 9. They show that participants with only 2 h of social media usage (or below this threshold) were classified as normal regardless of their age. Furthermore, participants aged 27 and below with less than 8 h of daily social media usage were still classified as normal. On the other hand, participants over 27 with an image contrast greater than 0.15 were classified as abnormal, meaning that they probably suffered from neck pain, regardless of the severity. In this regard, more details can be found in Table 9.

GLCM-based feature extraction combined with machine-learning (ML) algorithms is being applied to many medical applications such as mammograms for breast cancer detection, magnetic resonance imaging (MRI) for brain cancer detection, and unlimited non-medical applications. The numerical values of GLCM always depend on the pre-processing techniques used, such as thresholding, filtering types, and the angle rotation on the processed images. Contrast measures the special frequency of the image and is a different moment of GLCM; it can measure the variations between the highest and lowest values of the adjacent set of pixels. Therefore, in this study, contrast complied with DT branches of all ages and angle_1 very positively (Figure 9). To make the decision, it did not work alone with the pixels’ units; entropy was calculated with the significant parameters in the branches mentioned.

Further, the eight parameters calculated from 46 subjects were fed into the DT classifier with a training-test protocol of 66%–34%. The number of leaves was seven, and the size of the tree was thirteen. After evaluating the test split, the achieved accuracy and the F1-score were 94% and 0.94%, respectively. The result of the testing performance is illustrated in Table 8. This approach may be considered a new attempt to evaluate the influence of SM devices (i.e., with different neck postures) based on the achieved results.

Therefore, this research work could present the following contributions:

(1) New quantitative parameters have been proposed for studying or evaluating the effect of SM devices on their users’ neck muscles;
(2) The ability to use the DT rules increases the prediction of neck pain;
(3) The geometric measures of neck postures may increase the pain prediction;
(4) As is shown in Figure 10 and Table 10, the developed, MATLAB-based GUI implementing the mathematical GEP model can be employed as a reliable diagnostic tool to assist the orthopedic doctor;
(5) This research activity was approached objectively rather than subjectively. The number of input parameters received from retrospective X-ray images was seven (angle_1, angle_2, area, contrast, homogeneity, correlation, and energy), while the remaining parameters (age and H.mean—just two) were gathered through patient questionnaire (survey) forms. In percentage terms, compared to the total number of parameters considered (nine), they correspond to 77.7% and 22.2%, respectively.

It is important to note that the ETs were created using the ROC fitness function, and that six variable parameters were chosen (*d*_0_, *d*_2_ and *d*_6_–*d*_9_). In other words, the gender, H.mean, and GLCM characteristics were strongly implicated in the predictive mathematical model (as are reported in Equations (7)–(9)). The model was successful in determining the categorized limits (normal and abnormal classes) with an accuracy of 91.30%. GEP models have several advantages over other machine-learning techniques: they are closed-form solutions, making it easy for engineers and specialists to incorporate the models for solving various engineering issues, and GEP models are assumed more reliable than numerical and analytical solutions in neck-pain prediction, which are being used for the first time, to the best of our knowledge. Nonetheless, the accuracy is fairly acceptable, but increasing the samples and modifying the technique for acquiring new attributes (variables) that describe neck pain is fundamental for optimizing the method and obtaining higher-accuracy values.

Based on the scientific articles reported in Table 11, it is evident that all research works related to the effects of SM usage on neck muscles from 2008 to the present were conducted by using surveys (questionnaire only) [51,54,61]. Scientific approaches based on images analyses and related processing are quite limited; for example, D. David et al. analyzed the premature and inappropriate use of personal computers and cell phones and the related development of clinical symptoms commonly defined as “text-neck syndrome.” The authors studied the underlying causes and risk factors of musculoskeletal discomfort or pain by observing and processing the cervical–spine MRI images [62].

A few studies using angle measurement systems such as three-axis accelerometers were reported in [55,63,64,65,66]. The main disadvantages of these studies were that they became old and were not in sync with current SM devices (mainly smartphones) that have progressively developed to cover large populations of adults. On the other hand, the current studies from 2018 are all survey-questionnaire-based and do not objectively determine the pain neck symptoms [51,54,61]. Previously, the machine-learning applications have not been applied to this research area to the best of our knowledge. Thus, the quantitative analysis of neck postures combined with machine-learning techniques (e.g., decision trees) may improve the early prediction of neck-pain occurrences. This problem became a serious source of neck-pain prevalence during the COVID-19 pandemic. Patterns of online distance learning in pre-schools, schools, and universities push the authorities in charge to make new rules and guidelines for the long-term use of SM technology, especially by children and teens.

## 5. Conclusions

This research work developed a new approach for studying the influence of using SM devices or mini displays on neck-pain occurrences. This approach was performed on 46 retrospective X-ray images (subjects) through two measurement steps. First, three geometrical parameters were detected from the neck vertebral shapes associated with SM use for different time categories of three-hour bands. The first category (0–2 h) belonged to the normal cases (no pain). The next bands changed from 3 to more than 9 h and belonged to the abnormal cases (after pain). The parameters obtained in this step were angle_1, angle_2, and the area between them. In the second step, the GLCM was employed to extract four parameters depending on the image quality: contrast, homogeneity, correlation, and energy. Decision trees’ if–then rules were used to acknowledge which combination of the detected parameters may be applied to predict the abnormal occurrence of neck pain. The obtained classification accuracy of 94% and the F1-score of 0.95 both show that the DT machine-learning algorithm works well with fully satisfactory results [67].

The DT classifier proved its ability to handle unbalanced datasets, which are always a source of error in the healthcare domain. “CostSensitiveClassifier” parameters enable the classifier algorithm (Weka package) to regulate the over- and under-sampling methods. The DT algorithm demonstrated that the ratio of 5:1 between normal and abnormal participants did not have a significant impact on the classification accuracy. Improving such prediction and diagnosis techniques is extremely important for healthcare-service development. This research should be carried out to develop a new tool for measuring musculoskeletal neck-pain disease and symptoms.

The developments proposed in this research work will aid in the early prediction of neck pain, especially for people who use SM devices extensively. In this regard, based on the mathematical predictive model (Equations (7)–(9) discussed in the Section 2.8), a MATLAB-based graphical user interface (GUI) was developed, in which the orthopedic doctor or a generic user is able to select a specific X-ray neck image and enter the geometrical parameter (angles and area) values as well as the age, gender, and duration of SM usage related to the patient. Then, the MATLAB tool will be able to determine the other parameters/variables by means of GLCM method (as shown in Figure 10). As a result, the patient would be informed whether he/she is a candidate for neck pain or not. Furthermore, the user will be able to use the developed GUI directly on the smartphone to receive information in a timely manner.

## Figures and Tables

**Table 1:** Questionnaire (short survey) provided to patients during the orthopedic visit.

Question Number	Question Content				
1	Gender				
	Male	Female			
2	Age (years)				
	16–20	21–30	31–40	41–50	Above 51
3	Do you have diabetes?				
	Yes	No			
4	Do you suffer from high blood pressure?				
	Yes	No			
5	Do you browse social media?				
	Yes	No			
6	How many hours do you spend on social media a day?				
	<2 h	2–3 h	3–6 h	6–9 h or more	>9 h
9	Do you suffer from neck pain or neck tension?				
	Yes	No			
10	What type of pain do you face when you visit doctors? The doctor can help you understand the nature and type of pain.				
	Neck pain	Arthritis	Others		

**Table 2:** Pain location and the number of painful patients who participated in the study (total number of participants *N* = 46).

Pain Location	Number of Pained Patients Suffering from a Given Disease (Total Number of Patients *N* = 46)	Percentage of Participants Suffering from a Specific Disease Compared to the Total Number *N* = 46 of Participants (%)
Neck pain	38 (abnormal subjects)	82.60
Shoulder pain	9	19.56
Back pain	5	10.87
Arthritis	9	19.56
Bone pain	1	2.17
Diabetic	29	63.04
Blood pressure	17	36.95

**Table 3:** Demographics of the patients participating in the study (total number of participants *N* = 46).

Age of the Participants (Years)	Number of Participants	Percentage with Respect to the Total Number of Participants (*N* = 46)
(20–27)	4	8.70
(28–35)	21	45.65
(36–43)	11	23.91
(44–51)	8	17.39
(52–59)	2	4.35
Gender		
Males	19	41.30
Females	27	58.70
Subject Situation		
Normal (no perceived neck pain)	8	17.39
Abnormal (perceived neck pain)	38	82.60
Neck Flexion		
During the use of smartphones/handhelds, including for studying purposes (online).	46	100

**Table 4:** Statistics of the descriptive variables (i.e., age, H.mean, angle_1, angle_2, area, contrast, homogeneity, correlation, and energy) used in this study.

Parameter (Unit)	N	Minimum	Maximum	Mean		Std. Deviation
				Statistic	Std. Error	Statistic
Age (years)	46	24.00	58.00	36.89	1.22	8.28
H.mean (hour)	46	0.5	9.0	2.04	0.21	1.40
Angle_1 (degree)	46	38.29	101.89	79.56	2.04	13.81
Angle_2 (degree)	46	34.24	74.22	54.19	1.46	9.87
Area (mm^2^)	46	269.00	1305.00	1007.35	26.29	178.30
Contrast (pixels)	46	0.08	2.88	0.55	0.07	0.52
Homogeneity (pixels)	46	0.75	0.98	0.90	0.01	0.05
Correlation (pixels)	46	0.50	0.95	0.80	0.02	0.11
Energy (pixels)	46	0.16	0.76	0.41	0.02	0.13

**Table 5:** List of the GEP settings.

Parameter	Setting
Population size (P)	46
Fitness function	ROC
Dependent variable (Class)	1
Independent variables	10
Number of genes	5
Function set	$-,+,\times ,\;÷,\;x^{2},\;x^{3},\;x^{4},\;10^{x},\sqrt{x},\;\sqrt[{3}]{x},\;Exp\left(x\right),\;Ln\left(x\right)$
Maximum tree depth	10
Linking function between ETs	Multiplication

**Table 6:** List of the GEP variables.

Contributor to Class	Variable ID
Gender (male or female)	*d*_0_
Patient Age	*d*_1_
Time duration of social media use (H.mean)	*d*_2_
Angle_1	*d*_3_
Angle_2	*d*_4_
Area	*d*_5_
Contrast	*d*_6_
Homogeneity	*d*_7_
Correlation	*d*_8_
Energy	*d*_9_

**Table 7:** Correlation between the variables and responses related to the neck condition.

Parameter	Correlation Coefficient vs. Response
Gender	−0.017
Age	−0.270
Time Duration of social media usage (H.mean)	−0.253
Angle_1	−0.345
Angle_2	−0.139
Area	−0.369
Contrast	0.306
Homogeneity	−0.258
Correlation	−0.167
Energy	−0.223

**Table 8:** Summary of the testing results to evaluate the DT-classifier performance after oversampling.

Confusion Matrix		* Precision	* Recall	Accuracy	* F1-Score
13 (*TP*)	1 (*FN*)	0.96	0.94	94%	0.95
0 (*FP*)	2 (*TN*)				
**Cost Matrix**		**Cross Validation**	**Percentage Split**	**ROC Area**	**Total Number of Instances**
0	1	9-fold	66%	0.98	16
5	0				

* Weighted averages are calculated for normal and abnormal samples.

**Table 9:** Classification (IF-THEN) rules obtained by the C4.5 algorithm for the completed DT shown in Figure 9.

1	*Social_Media_use* “=1_hours” $\overset{THEN}{\to }$ *Diagnose* “Normal”
2	*Social_Media_use* “=2_hours” $\overset{THEN}{\to }$ *Diagnose* “Normal”
3	*Social_Media_use* “=8_10_hours” AND *Age* “<=27” $\overset{THEN}{\to }$ *Diagnose* “Abnormal”
4	*Social_Media_use* “<8_10_hours” AND *Age* “<=27” $\overset{THEN}{\to }$ *Diagnose* “Normal”
5	*Social_Media_use* “>2_hours and <8_10_hours” AND *Age* “>27” *AND Contrast* “<=0.1455” *AND angle_1* “<=72.42” $\overset{THEN}{\to }$ *Diagnose* “Normal”
6	*Social_Media_use* “>2_hours” AND *Age “>27” AND Contrast “<=0.1455” AND angle_1 “>72.42”* $\overset{THEN}{\to }$ *Diagnose* “Abnormal”
7	*Social_Media_use* “>2_hours” AND *Age* “>27” *AND Contrast “>0.1455”* $\overset{THEN}{\to }$ *Diagnose* “Abnormal”

**Table 10:** Results of the implementation of the GEP prediction model on all 46 patients for predicting the neck-pain condition. As for the predicted class column, value 1 indicates the normal class (no neck pain for H value > 0.5), value 0 the abnormal class (perceived pain for H value < 0.5). The meanings of the parameters di (i = 0, 1, …, 8) are reported in Section 2.8 (Table 6), related to the mathematical GEP model developed for the neck-pain prediction. The indications given in the column “Class Matching” refer to the definitions provided in the Section 3.2 (on page 18).

Patient N.	Gender *d*_0_	Age *d*_1_	H.mean *d*_2_	Angle_1 *d*_3_	Angle_2 *d*_4_	Area *d*_5_	Contrast *d*_6_	Homo-geneity *d*_7_	Correlation *d*_8_	Energy *d*_9_	Input Class	GEP Model (H)	Predicted Class	Class Matching
1	Male	29	3.5	72.42	41.35	1103	0.297	0.914	0.907	0.306	Abnormal (0)	0.006	Abnormal (0)	OK (TN)
2	Male	30	1.5	52.06	60.84	896	0.765	0.836	0.770	0.230	Abnormal (0)	0.028	Abnormal (0)	OK (TN)
3	Male	32	9.0	79.65	71.05	1012	1.293	0.854	0.797	0.407	Abnormal (0)	0.001	Abnormal (0)	OK (TN)
4	Male	35	3.5	62.86	36.87	986	0.451	0.913	0.758	0.392	Abnormal (0)	0.004	Abnormal (0)	OK (TN)
5	Male	35	7.5	59.96	59.1	1279	0.577	0.881	0.879	0.382	Abnormal (0)	0.001	Abnormal (0)	OK (TN)
6	Male	38	9.0	90.01	50.04	1070	0.550	0.899	0.846	0.430	Abnormal (0)	0.001	Abnormal (0)	OK (TN)
7	Male	41	6.0	87.07	47.67	1009	1.300	0.839	0.653	0.399	Abnormal (0)	0.001	Abnormal (0)	OK (TN)
8	Male	43	3.5	65.4	47.12	993	0.168	0.945	0.935	0.358	Abnormal (0)	0.028	Abnormal (0)	OK (TN)
9	Male	46	9.0	53.61	56.95	941	0.499	0.930	0.706	0.681	Abnormal (0)	0.001	Abnormal (0)	OK (TN)
10	Male	51	0.5	70.13	46.61	939	0.376	0.899	0.780	0.386	Abnormal (0)	0.001	Abnormal (0)	OK (TN)
11	Male	54	0.5	75.32	40.08	966	0.188	0.962	0.849	0.760	Abnormal (0)	0.001	Abnormal (0)	OK (TN)
12	Male	58	0.8	38.29	38.81	1123	0.310	0.941	0.857	0.602	Abnormal (0)	0.001	Abnormal (0)	OK (TN)
13	Female	25	9.0	89.08	68.77	1170	0.679	0.875	0.688	0.290	Abnormal (0)	0.001	Abnormal (0)	OK (TN)
14	Female	27	9.0	86.97	52.87	1305	0.300	0.930	0.810	0.412	Abnormal (0)	0.001	Abnormal (0)	OK (TN)
15	Female	28	9.0	77.25	74.22	1005	1.128	0.809	0.643	0.194	Abnormal (0)	0.001	Abnormal (0)	OK (TN)
16	Female	28	8.0	88.87	71.37	414	1.926	0.791	0.609	0.158	Abnormal (0)	0.001	Abnormal (0)	OK (TN)
17	Female	28	9.0	93.01	69.41	921	0.395	0.912	0.754	0.473	Abnormal (0)	0.001	Abnormal (0)	OK (TN)
18	Female	29	9.0	93.61	55.29	1105	0.675	0.886	0.683	0.373	Abnormal (0)	0.001	Abnormal (0)	OK (TN)
19	Female	30	7.0	101.89	68.45	917	0.149	0.951	0.934	0.494	Abnormal (0)	0.004	Abnormal (0)	OK (TN)
20	Female	31	7.0	84.22	53.13	1260	0.147	0.952	0.941	0.451	Abnormal (0)	0.003	Abnormal (0)	OK (TN)
21	Female	32	6.0	89.95	62.34	1094	0.595	0.893	0.739	0.350	Abnormal (0)	0.001	Abnormal (0)	OK (TN)
22	Female	32	7.5	99.41	54.11	988	0.364	0.904	0.775	0.357	Abnormal (0)	0.001	Abnormal (0)	OK (TN)
23	Female	32	9.0	94.01	58.35	1111	0.566	0.902	0.770	0.352	Abnormal (0)	0.001	Abnormal (0)	OK (TN)
24	Female	33	5.6	91.76	58.65	1010	0.148	0.954	0.944	0.440	Abnormal (0)	0.016	Abnormal (0)	OK (TN)
25	Female	35	5.5	98.58	62.61	1064	0.817	0.866	0.701	0.272	Abnormal (0)	0.001	Abnormal (0)	OK (TN)
26	Female	35	5.5	82.75	49.65	1084	0.503	0.887	0.761	0.278	Abnormal (0)	0.001	Abnormal (0)	OK (TN)
27	Female	35	7.0	90.62	51.84	1116	0.358	0.902	0.794	0.333	Abnormal (0)	0.001	Abnormal (0)	OK (TN)
28	Female	36	4.5	89.32	62.01	1159	0.247	0.950	0.789	0.593	Abnormal (0)	0.114	Abnormal (0)	OK (TN)
29	Female	37	5.5	89.31	60.28	1023	0.123	0.953	0.931	0.511	Abnormal (0)	0.093	Abnormal (0)	OK (TN)
30	Female	42	1.5	85.57	42.02	1080	0.533	0.889	0.753	0.385	Abnormal (0)	0.001	Abnormal (0)	OK (TN)
31	Female	42	5.5	76.91	69.09	1057	0.605	0.872	0.778	0.307	Abnormal (0)	0.001	Abnormal (0)	OK (TN)
32	Female	46	0.8	77.76	48.61	848	0.418	0.923	0.826	0.397	Abnormal (0)	0.001	Abnormal (0)	OK (TN)
33	Female	48	1.5	90.26	46.14	1050	0.158	0.946	0.939	0.419	Abnormal (0)	0.001	Abnormal (0)	OK (TN)
34	Female	50	7.0	80.89	50.87	987	0.156	0.948	0.939	0.417	Abnormal (0)	0.002	Abnormal (0)	OK (TN)
35	Male	37	4.5	57.05	56.14	1065	0.142	0.953	0.931	0.470	Normal (1)	0.694	Normal (1)	OK (TP)
36	Male	39	2.5	60.48	46.15	1044	0.170	0.948	0.940	0.395	Normal (1)	1.000	Normal (1)	OK (TP)
37	Male	41	3.0	67.85	50.57	912	0.146	0.952	0.937	0.459	Normal (1)	1.000	Normal (1)	OK (TP)
38	Male	45	1.5	85.49	58.04	1143	0.265	0.945	0.846	0.661	Normal (1)	1.000	Normal (1)	OK (TP)
39	Female	24	2.5	79.82	50.52	1055	0.822	0.868	0.711	0.432	Normal (1)	0.691	Normal (1)	OK (TP)
40	Female	27	4.0	79.57	48.55	269	2.881	0.751	0.505	0.170	Normal (1)	1.000	Normal (1)	OK (TP)
41	Female	30	1.5	64.12	51.31	888	0.837	0.839	0.652	0.237	Normal (1)	0.940	Normal (1)	OK (TP)
42	Female	36	2.0	79.64	54.06	1067	0.472	0.898	0.764	0.497	Normal (1)	1.000	Normal (1)	OK (TP)
43	Male	35	7.0	77.17	52.06	1075	0.080	0.977	0.946	0.727	Abnormal (0)	0.994	Normal (1)	Wrong (FP)
44	Male	48	2.5	90.47	64.04	985	0.182	0.937	0.931	0.365	Abnormal (0)	1.000	Normal (1)	Wrong (FP)
45	Male	50	1.5	82.09	34.24	789	1.321	0.845	0.669	0.337	Abnormal (0)	1.000	Normal (1)	Wrong (FP)
46	Female	32	1.5	77.08	40.72	961	0.423	0.910	0.771	0.385	Abnormal (0)	1.000	Normal (1)	Wrong (FP)

Note: the 1-value in the class column (input and predicted class) indicates the normal (no perceived pain) cases, whereas the 0 value indicates the abnormal (perceived pain) ones. A class matching value of “OK” means that the “Input Class” normal (value 1) is correctly predicted as normal and defined as True Positive (TP), or that the “Input Class” abnormal (value 0) is correctly predicted as Abnormal and defined as True Negative (TN)

**Table 11:** Comparison of this work with similar scientific works reported in the literature analyzing the impact of using the SM devices on neck-pain occurrence.

First Author, Ref. n., Year	Method Survey-Based (Subjective) or Quantitative (Objective)	Samples (Male/Female)	Evaluation Approach		Parameters #	Target of Study
			Machine-Learning Type	Statistics		
F. Al-Hadidi [51], 2019	Online Questionnaire (Subjective)	500	None	Significant test (*p* < 0.001)	Two: duration, neck pain	To investigate the association between neck pain and the duration of device use, taking into consideration gender, age, and the most frequent position in which students use their devices.
S. A. Rahman [61], 2020	Questionnaire (Subjective)	300	None	Significant test (*p* < 0.003)	Four: gender, age, university campus, academic year	To investigate the effects of SM use on health and academic performance of students at Sharjah University
D. David [62], 2021	MRI of the cervical spine (Objective)	1 case reported	None	None	One: neck flexion angle	To analyze the new phenomenon of the “text-neck syndrome”
S. Lee [63], 2014	Three-dimensional motion capture system (Objective)	18	None	Significant test (*p* < 0.05)	One: Head flexion angle	To quantitatively assess the amount and range of head flexion of smartphone users
L. Straker [64], 2008	Three-dimensional posture and muscle activity measurement (Objective)	18	None	Significant test (*p* < 0.001)	Three: working in desktop, tablet, and paper conditions and measuring the head angle	To compare the posture and muscle activity of children using a tablet computer to the posture and muscle activity of children using a desktop computer and paper technology.
E. Gustafsson [65], 2011	Angle measuring system (Objective)	56	None	Significant test (*p* < 0.05)	Three: symptomatic, asymptomatic, head angle	To investigate differences in technique between young adults with and without musculoskeletal symptoms when using a mobile phone for texting, and differences in muscle activity and kinematics between different texting techniques.
H. Ping Chiu [66], 2015	Angle measuring system (Objective)	30	Electromyography measurement	Significant test (*p* < 0.000)	Three: tilt angle, task type, neck muscle activity	To investigate the musculature load and comfort perception of the engaged upper extremity for three angles of viewing, and common task types performed at a computer workstation.
A. Widhiyanto [54], 2018	Questionnaire (Subjective)	979	None	Significant test (*p* < 0.05)	Two: Duration, Class	To analyze the effect of duration of smartphone use on neck pain.
H. Lee [55], 2013	Three-axis accelerometer (Objective)	12	Built-in sensors, front- faced camera, three-axis accelerometer	None	One: angle	To monitor the posture of smartphone users by built-in sensors.
This work	Angles measurement and X-ray images analysis (Objective)	46	Decision tree. Classification accuracy = 94%	Significant test	Nine: age, H.mean, angle_1, angle_2, area, contrast, homogeneity, correlation, energy	To detect neck pain by: (i) Using two angles and the area between them instead of just one angle; (ii) Applying the image statistical features (GLCM) in the same procedure; (iii) Utilizing DT algorithm; (iiii) Using a MATLAB- based GUI to help the doctor in the diagnosis of neck pain in an immediate way.

## Data Availability

Data from our study are available upon request.

## Supplementary Materials

The following supporting information can be downloaded at: https://www.mdpi.com/article/10.3390/jimaging9010014/s1, Video S1: MATLAB-based application for neck-pain prediction.mp4.

## Abbreviations

List of the abbreviations and related meaning.

Acronym	Meaning and Definition
Angle_1	The neck-flexion angle between the global horizontal and the vector pointing from C7 vertebra to the occipito-cervical joint.
Angle_2	The neck-flexion angle between the global horizontal and the vector pointing from C1 vertebra to the occipito-cervical joint.
C1, C7	Neck vertebrae
C4.5	DT algorithm named C4.5
DT	Decision tree
E	Entropy
FFT	Fast Fourier transform
FN	False negative
FP	False positive
GLCM	Gray-level co-occurrence matrix (GLCM)
IG	Information gain
ML	Machine learning
NDI	Neck disability index
NP	Neck pain
RMSE	Root mean square error
ROC	Receiver operating characteristic
SM	Social media
Std. Error	Standard error
STDev	Standard deviation
TN	True negative
TP	True positive
VAS	Visual analogue scale
WHO	World health organization
